# Dataset on resource allocation and usage for a private cloud

**DOI:** 10.1016/j.dib.2026.112514

**Published:** 2026-01-29

**Authors:** Paola Marques, Mariana Mendes, Thiago Emmanuel Pereira, Giovanni Farias

**Affiliations:** Department of Computing and Systems, Federal University of Campina Grande, Campina Grande, Brazil

**Keywords:** IaaS, Infrastructure monitoring, Workload characterization, OpenStack

## Abstract

While public cloud providers dominate the commercial landscape, private clouds are widely adopted by academic and research institutions to meet specific governance and operational requirements. There are multiple available datasets about resource usage of public clouds; however, datasets capturing usage patterns in private clouds remain scarce, which limits research in this area. This work presents a dataset comprising over 64 million records collected from a private OpenStack-based cloud operated by the Distributed Systems Laboratory at the Federal University of Campina Grande, Brazil. Data was continuously gathered over nearly twelve months (May 23, 2024 to May 16, 2025), periodically querying OpenStack APIs and monitoring services every five minutes. The dataset captures different aspects of the infrastructure, allocation quotas, user-to-project associations (as OpenStack groups users into projects), server (virtual machines) specifications, and resource utilization for users and projects. Entries are timestamped, enabling temporal analyses of system dynamics. Sensitive attributes, such as user names, project names, IP addresses, and server names were protected, leaving only system-generated UUIDs. By offering a detailed, time-stamped, view of a private cloud, this dataset provides a valuable resource for cloud computing research, helping to bridge the gap in publicly available datasets from non-commercial cloud environments. The dataset is valuable not only for academic institutions but also for companies considering cloud repatriation.

Specifications Table

**Table:** 

Subject	Computer Science
Specific subject area	Cloud computing, Quota management, Resource Usage Patterns
Type of data	Tables in CSV files
Data collection	Data was collected using a monitoring tool developed by the Distributed Systems Laboratory (LSD/UFCG). The service runs on a server in the LSD private cloud and periodically queries OpenStack APIs (for metadata on projects, users, servers, quotas, flavors) and Prometheus endpoints (for resource utilization metrics obtained from the libvirt exporter). Collection was scheduled every five minutes, with results stored in a PostgreSQL database. Sensitive details were not retrieved, leaving only system identifiers (UUIDs). No further normalization was applied; raw records were exported monthly as CSV files.
Data source location	Department of Computing and Systems, Federal University of Campina Grande, Campina Grande, Brazil
Data accessibility	Repository name: Mendeley Data Data identification number: 10.17632/trvb5k4×5m.1 Direct URL to data: https://data.mendeley.com/datasets/trvb5k4×5m/1 Instructions for accessing these data: No restriction to data accessibility. License: Creative Commons Attribution 4.0 International
Related research article	None.

## 1 Value of the Data

• The dataset allows workload characterization, demand forecasting, and performance prediction. It enables the training and evaluation of machine learning models for forecasting CPU and memory demand, supporting demand prediction, identification of correlated workload patterns across servers, and temporal correlation analysis. These capabilities help improve auto-scaling decisions, reduce resource overprovisioning and performance degradation.
• The dataset is well suited for research on how quota constraints influence user behavior and resource distribution fairness in cloud environments. Its project-level quota and utilization information enable researchers to study quota adherence patterns, investigate potential quota attribution strategies, and analyze resource contention dynamics. In addition, the explicit attribution of resource utilization to projects and users supports research on the evaluation of billing, metering, and accounting models over extended time periods.
• The dataset also supports research on infrastructure efficiency, cost optimization, and long-term capacity planning in private cloud environments. Its historical utilization traces enable the identification of persistently idle or underutilized resources, allowing the quantification of potential savings from automated shutdown or suspension policies. By correlating allocated resource specifications (vCPU and memory) with observed utilization peaks, researchers can derive right-sizing recommendations and identify systematically over-provisioned configurations. Furthermore, the extended temporal coverage enables studies on capacity planning and server consolidation, allowing the modeling of infrastructure requirements and efficiency gains needed to meet future demand.
• As the dataset is extracted directly from production OpenStack monitoring, it provides authentic traces of scheduler behavior, memory management dynamics, and tenant isolation mechanisms in a real deployment. This makes it well suited for evaluating OpenStack placement and scheduling strategies by analyzing how workloads are distributed across hosts and assessing fairness and resource utilization. In addition, the dataset enables the evaluation of new scheduling policies under realistic private-cloud conditions. In contrast to the common practice of relying on public-cloud traces or synthetic workloads, these data reflect the operational constraints, user behaviors, and resource contention patterns of a production academic OpenStack environment.
• The dataset is particularly applicable to environments characterized by multi-tenancy, quota-based resource management, and long-running or research-oriented workloads. Conversely, it is not intended to represent large-scale public cloud platforms or workloads primarily characterized by extreme elasticity, rapid scaling, and short-lived execution patterns, such as web-scale SaaS applications, microservice-based architectures, or serverless computing environments. Nevertheless, the dataset is particularly useful for supporting system research on hypervisors, applied to both private and public clouds. For example, to support the evaluation and design of new management strategies, including memory hotplugging and ballooning, processes, and I/O scheduling.

## 2 Background

Public cloud datasets such as Google Cluster [1], Alibaba Trace [2] and Azure [3], have significantly contributed to cloud computing research, supporting studies on scheduling, workload modelling, demand forecasting, and infrastructure optimization [[8], [9], [10]]. However, publicly available dataset representing private cloud environments usage remain scarce. Existing work frequently depends on synthetic or simulated workloads [[4], [5], [6]], while operational differences between public and private clouds (such as resource allocation strategies, logical organization, and user behavior) limit the generalizability of findings across different contexts.

The dataset presented in this article addresses this gap by providing comprehensive, time-stamped records from an OpenStack-based private cloud used in production. Unlike commercial datasets, this collection captures and organises custom data related to the dynamics of a private cloud. These traits include server provisioning, quota enforcement, association of users with workforce teams, and the impact of server sizing.

By offering this level of detail, the dataset enables research in system planning, anomaly detection, server placement, scheduling, resource overcommitment and consolidation, and hypervisor design. It represents a valuable resource for researchers, engineers, and system administrators aiming to analyze, model, and optimize private cloud infrastructures, not only in educational and research contexts but also for companies moving from public to private cloud environments.

## 3 Data Description

The dataset comprises over 64 million entries extracted from a database that stored monitoring records collected from a private cloud through a collection service. It provides a detailed view of the environment by capturing projects, users, flavors and servers. Each one of these entities is better described in the following subsections. Records include a timestamp in UNIX epoch seconds, enabling analyses spanning nearly one year of data (from May 23, 2024 to May 16, 2025). The identifiers used throughout the dataset - *project_id, user_id, server_id*, and *flavor_id -* are stored as UUIDs, which are randomly generated unique identifiers automatically assigned by the OpenStack environment to ensure consistent cross-referencing among tables. This dataset has been published in Mendeley Data [7], preserving the original structure of the source database, and is accompanied by a README file describing the relationships between tables.

**Fig. 1:**
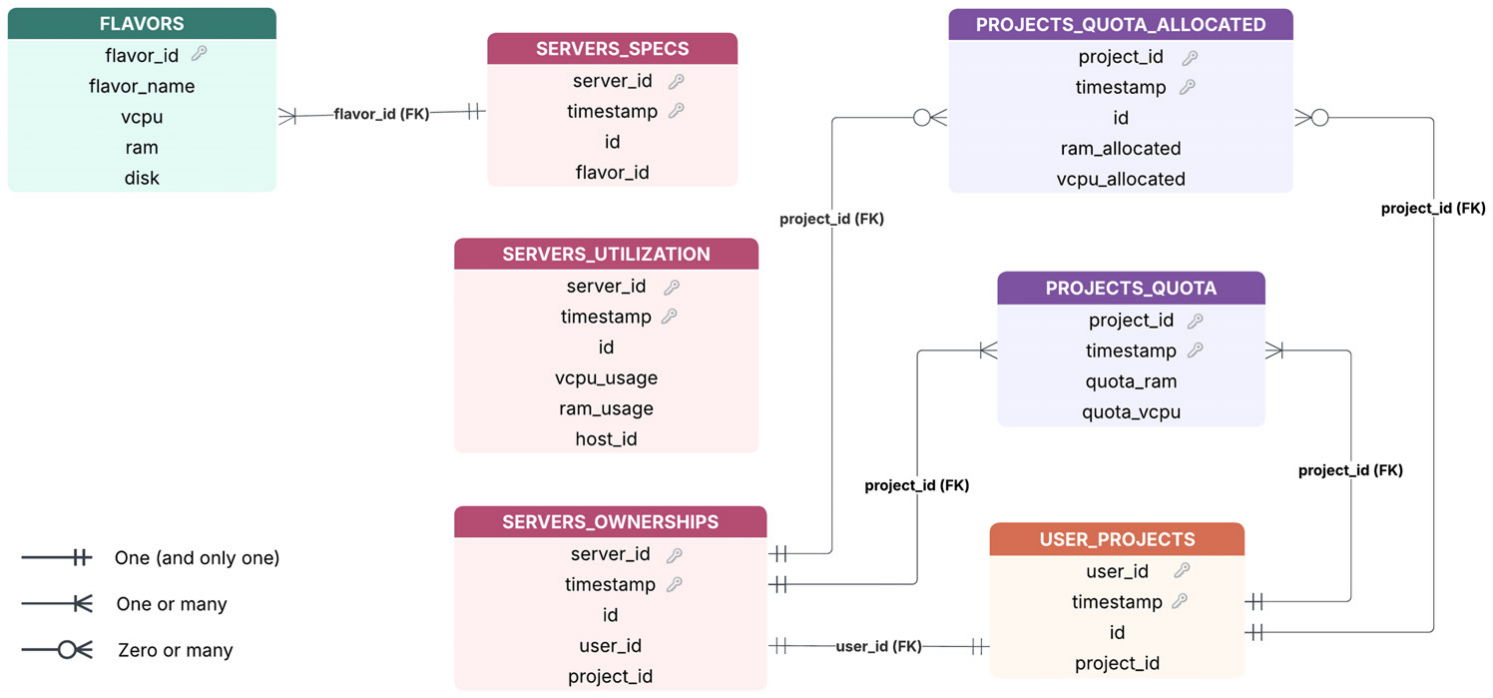
Database schema. The figure illustrates the conceptual relational structure of the database and its main entities. Tables with a timestamp attribute represent time series associated with a given identifier over time.

An overview of these tables is provided below in Fig. 1, showing their relationships and structure.

### 3.1 Projects

In the OpenStack environment, a project represents an organizational unit that groups users and servers, and serves as the primary scope for resource allocation and policy enforcement. Quotas and other resource management constraints are defined at the project level and directly influence how resources are provisioned and consumed.

Project information is represented by two tables: *projects_quota* (see Table 1) and *projects_quota_allocated* (see Table 2). The *projects_quota* table records the quota configuration assigned to each project over time. Because quota changes occur infrequently, entries in this table are only created when a quota update takes place, such as changes to vCPU, memory, or disk limits. This table can therefore be used as a reference to retrieve the quota configuration of a project at any given point in time.

**Table 1:** Resource quota allocations per project (RAM and vCPU).

Column	Type	Description
timestamp	bigint	Collection time in UNIX epoch seconds.
project_id	text	Unique project identifier (UUID).
quota_ram	bigint	RAM quota available to the project (MB).
quota_vcpu	bigint	vCPU quota available to the project.

**Table 2:** Utilization of allocated quotas per project (RAM and vCPU).

Column	Type	Description
Id	bigint	Unique row identifier.
timestamp	bigint	Collection time in UNIX epoch seconds.
project_id	text	Unique project identifier (UUID).
ram_allocated	bigint	RAM quota allocated per project (MB).
vcpu_allocated	bigint	vCPU quota allocated per project.

The *projects_quota_allocated* table captures the amount of quota currently allocated to servers within each project. By analyzing this table over time and combining it with the corresponding quota limits from *projects_quota*, researchers can derive metrics such as the percentage of quota consumed, remaining available capacity, and temporal trends in resource allocation.

**Fig. 2:**
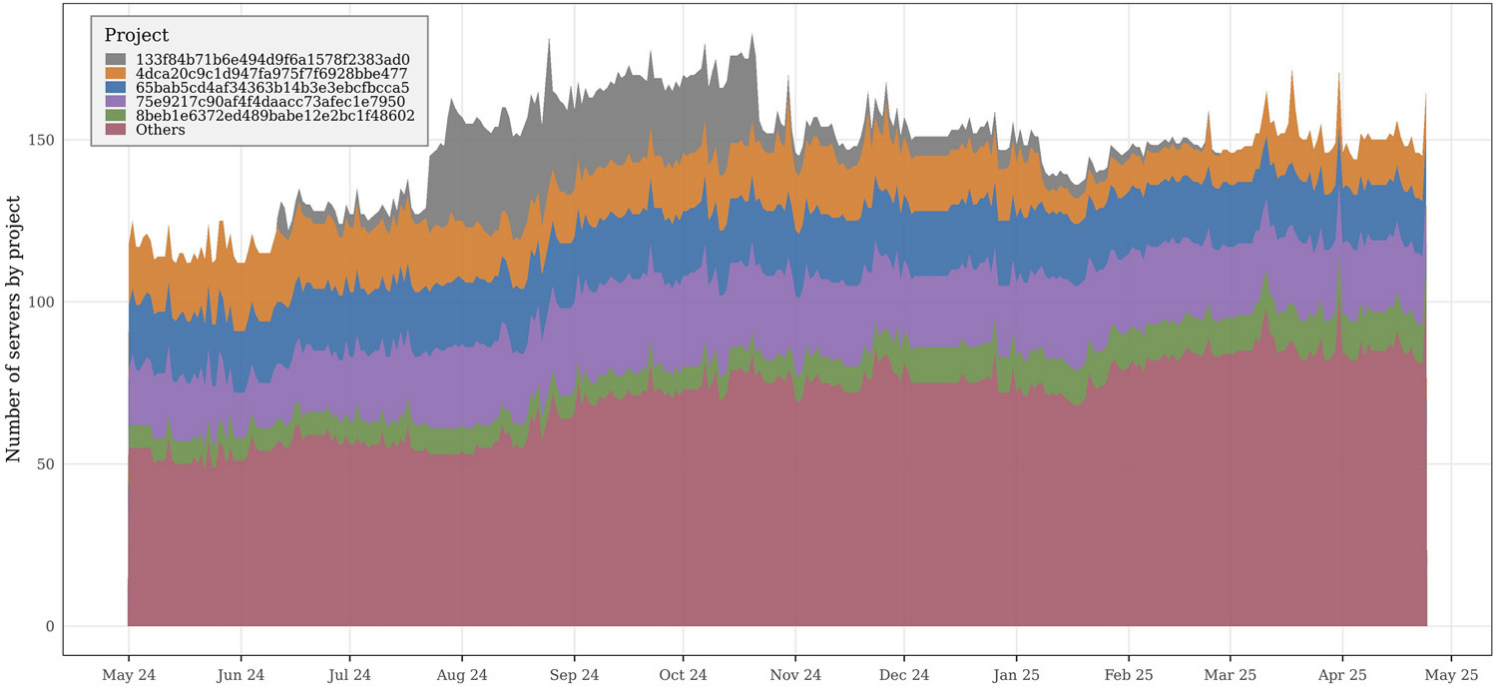
Daily number of servers per project. The figure presents both the total number of active servers and how this volume is distributed across projects.

When combined with other tables, such as *servers_ownerships* (see Table 5), the dataset enables cross-entity analyses linking projects, users, and servers. For example, joining these tables makes it possible to compute project-level server distributions or to identify projects with a high concentration of active servers, as illustrated in Fig. 2, which presents a ranking of projects by the number of associated servers.

Overall, the project quota tables support analyses focused on quota-aware resource management in cloud environments. Typical use cases can include studies on identification of potential quota gaming strategies (e.g. when a user joins multiple projects to harness the quota of the projects), evaluation of quota policy effectiveness, and fairness analysis across projects with different allocation limits.

### 3.2 Users

This subsection describes the data related to users in the cloud environment. In OpenStack, a user represents an authenticated cloud consumer who can be a member of one or more projects and is authorized to create and manage servers within the limits defined by project-level policies. Each user is identified by a unique identifier. User names are intentionally omitted from the dataset to preserve anonymity.

User information is represented by a single table, *user_projects* (see Table 3), which records the association between users and projects over time. Because user memberships may change, entries in this table include timestamps to get when a user becomes associated with or dissociated from a given project. This temporal information allows the table to be used as a reference for reconstructing the relationship between project and user.

**Table 3:** User-to-project memberships.

Column	Type	Description
id	bigint	Unique row identifier.
timestamp	bigint	Collection time in UNIX epoch seconds.
user_id	text	Unique user identifier (UUID).
project_id	text	Unique project identifier (UUID).

**Fig. 3:**
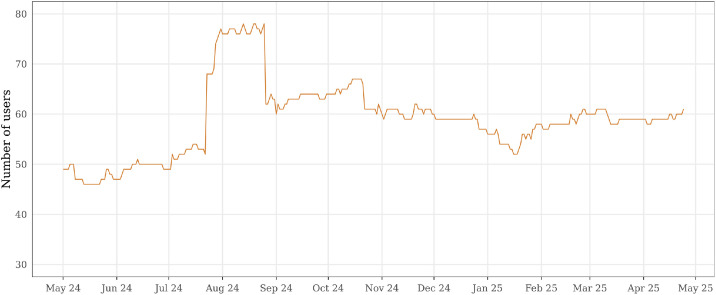
Daily number of active users with allocated servers in the private cloud, showing the temporal evolution of users actively consuming server resources over the entire observation period.

When analyzed in isolation, the *user_projects* table enables descriptive analyses of user population dynamics, such as tracking the number of active users per project over time or identifying periods of membership churn, as illustrated in Fig. 3. These analyses provide context on how users are distributed across organizational units and how project participation evolves.

When combined with other tables, the *user_projects* table supports multi-dimensional analyses. For example, joining it with *servers_ownerships* via the shared *user_id* enables the computation of daily active users based on server activity, supporting analyses of workload distribution across users or aggregation of resource consumption patterns at the user level. Similarly, integrating user and project membership with project tables allows researchers to study how users operate under different quota policies.

### 3.3 Servers

This subsection describes the data related to servers, which in this dataset correspond to virtual machines instantiated in the cloud infrastructure. Each server is uniquely identified by the *server_id* field, a UUID automatically assigned by OpenStack. To preserve user privacy and infrastructure security, sensitive attributes such as server names are not included in the dataset. Server information is distributed across four main tables: *servers_specs* (see Table 4), *servers_ownerships* (see Table 5), *servers_utilization* (see Table 6), and *servers_lifecycle* (see Table 7), which together describe server configuration, ownership context, resource utilization, and server lifecycle.

**Table 4:** Server association with their flavor assignment.

Column	Type	Description
id	bigint	Unique row identifier.
timestamp	bigint	Collection time in UNIX epoch seconds.
server_id	text	Unique server identifier (UUID).
flavor_id	text	Unique flavor identifier (UUID).

**Table 5:** Server ownership: user and project mapping.

Column	Type	Description
id	bigint	Unique row identifier.
timestamp	bigint	Collection time in UNIX epoch seconds.
server_id	text	Unique server identifier (UUID).
user_id	text	Unique user identifier (UUID).
project_id	text	Unique project identifier (UUID).

**Table 6:** Server resource utilization metrics (vCPU %, RAM %, host).

Column	Type	Description
id	bigint	Unique row identifier.
timestamp	bigint	Collection time in UNIX epoch seconds.
server_id	text	Unique server identifier (UUID).
vcpu_utilization	float	vCPU utilization ( %).
ram_utilization	float	Memory (RAM) utilization ( %).
host_id	text	Compute host identifier.

**Table 7:** Server lifecycle registry.

Column	Type	Description
server_id	text	Unique server identifier (UUID).
created_at	bigint	Server creation time in UNIX epoch seconds.
ended_at	bigint	Server termination time in UNIX epoch seconds, when applicable.

The *servers_specs* table records the hardware configuration assigned to each server over time, referencing the flavor identifier associated with that configuration. Because flavor identifiers are opaque, meaningful interpretation of this table requires joining it with the *flavors* table (See Table 8), which explicitly defines allocated vCPU cores, memory, and disk capacity. This temporal structure allows researchers to track configuration changes and analyze how resource allocations evolve throughout a server’s lifetime.

**Table 8:** Flavor specifications.

Column	Type	Description
flavor_id	text	Unique flavor identifier (UUID).
flavor_name	text	Name of the flavor.
vcpu	float	Number of vCPUs defined in the flavor.
ram	float	RAM capacity defined in the flavor (MB).
disk	float	Disk capacity defined in the flavor (GB).

The *servers_ownerships* table captures the association between servers, users, and projects, indicating which user instantiated a given server and under which project it operates. When combined with project and user tables, this structure enables analyses of server distribution across organizational units, user activity concentration, and project-level infrastructure usage.

**Fig. 4:**
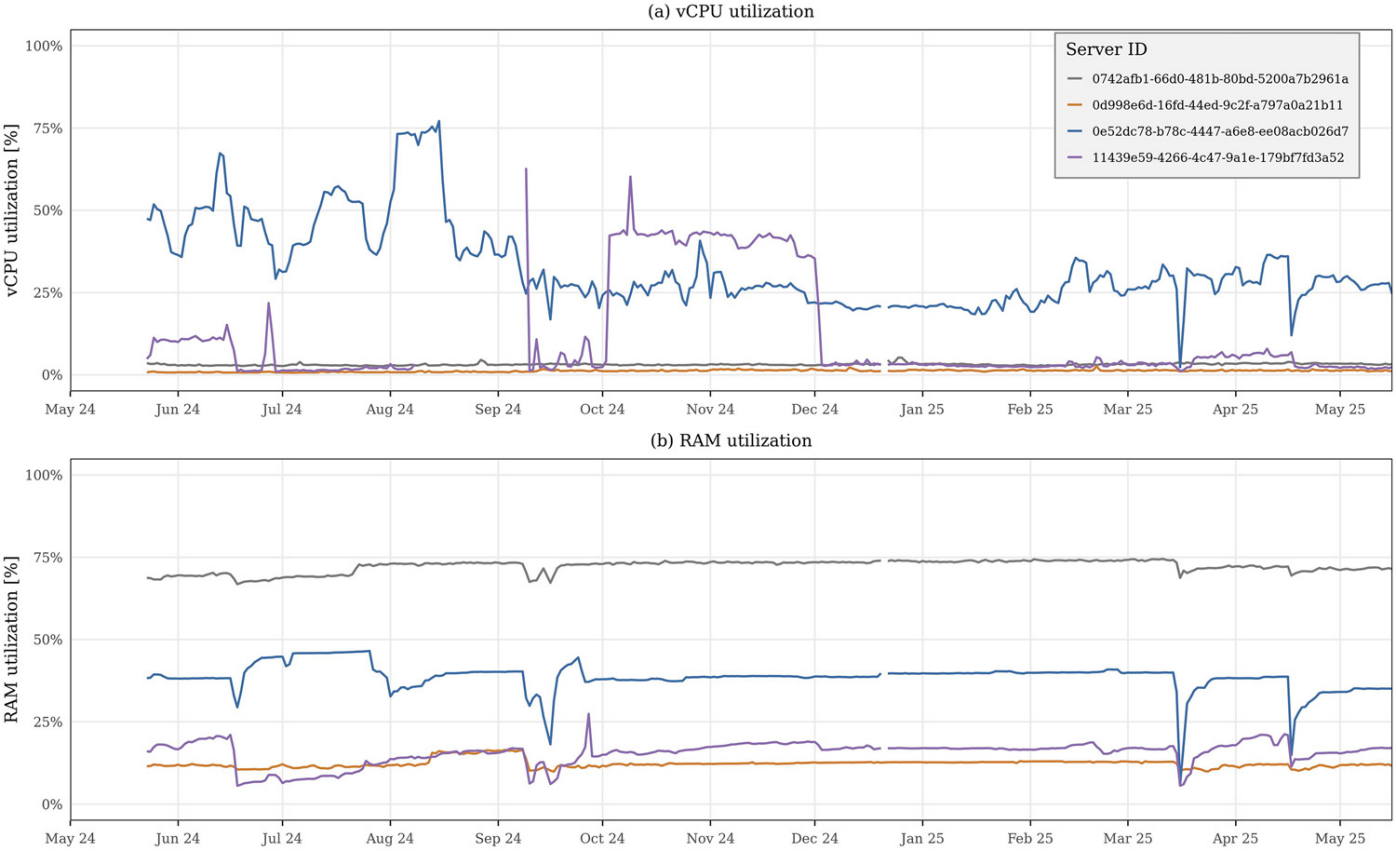
Daily average vCPU and RAM utilization of four active servers over the observation period, illustrating heterogeneous resource utilization patterns across servers.

The *servers_utilization* table is the central component of the dataset and provides fine-grained time-series measurements of vCPU and memory utilization for each server. These measurements enable a wide range of analyses, from aggregate infrastructure studies to detailed per-server behavior characterization. When analyzed independently, this table supports descriptive evaluations of overall datacenter utilization trends, such as average and peak vCPU and memory utilization over time, as illustrated in Fig. 4.

The *servers_lifecycle* table records the start and end timestamps for each server, enabling analyses of server creation dynamics and the measurement of individual server lifetimes over the monitored period.

When combined with *servers_specs*, the *servers_utilization* data enables researchers to contrast allocated and observed resource utilization, supporting analyses of over-provisioning and underutilization. Aggregating utilization metrics by project, user, or flavor enables the identification of systematic usage patterns. The longitudinal nature of the data further supports workload modeling, demand forecasting, and the evaluation of capacity planning and consolidation strategies.

By integrating *servers_utilization* with *servers_ownerships* and configuration metadata through shared identifiers (s*erver_id*), the dataset enables studies on fairness, resource efficiency, and policy design without requiring access to infrastructure details. This relational structure allows each table to be used independently for exploratory analysis or jointly for more comprehensive and multi-dimensional investigations.

### 3.4 Flavors

This subsection describes the data related to flavors in the cloud environment. In OpenStack, a flavor represents a predefined hardware configuration that specifies the virtual resources allocated to a server, such as the number of vCPUs, memory capacity, and disk size. Flavors define the resource profile available to users when provisioning servers.

Flavor information is stored in a single table, *flavors* (see Table 8), which records the technical specifications for each available hardware configuration. Flavor names are retained as descriptive attributes to facilitate interpretation and comparison across configurations.

When considered on their own, the *flavors* table enables descriptive analyses of the hardware configurations offered by the cloud, such as examining the diversity of available resource profiles or comparing capacity characteristics across flavors. This perspective provides insight into how the infrastructure exposes different compute options to users.

In combination with other tables, flavor data support more detailed analyses of server behavior and resource utilization. By joining the *flavors* table with *servers_specs* through the shared flavor identifier, servers can be associated with their corresponding hardware configurations.

**Fig. 5:**
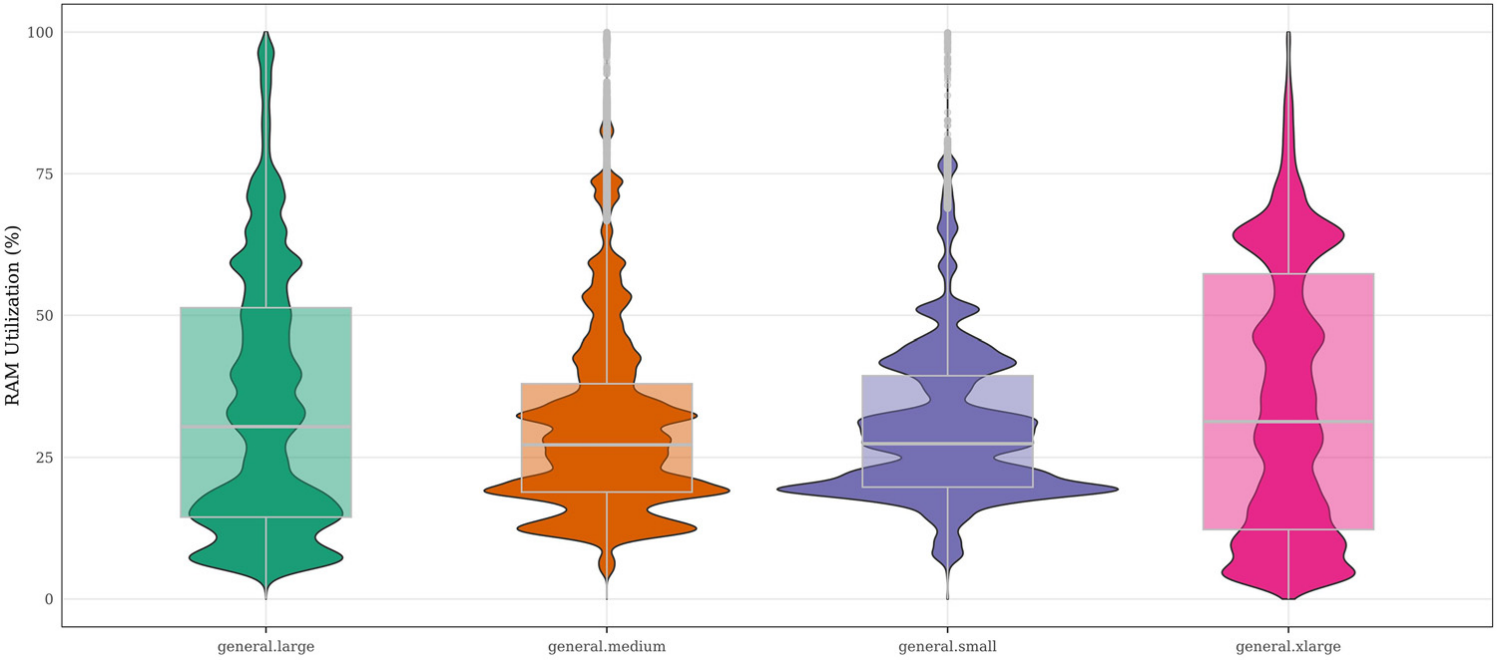
Distribution of RAM utilization ( %) for servers grouped by flavor name. The figure illustrates RAM usage patterns for servers according to their flavor definitions.

Further integration with the *servers_utilization* table allows resource consumption metrics to be grouped by flavor, enabling comparisons of usage patterns across hardware profiles. Fig. 5 illustrates this integration by presenting the distribution of RAM utilization percentages for servers grouped by flavor name, providing a basis for evaluating over- and underutilization patterns across different hardware configurations. This data supports research decisions related to deciding the flavor offering. That is, how to decide the set of flavors (and their specification) to be offered in the cloud to reduce waste, given the actual demand for resources and resource utilization. Also, this data supports the research on bin-packing heuristics to reduce host fragmentation, including the effect of flavors specs into the packing of servers into hosts.

Although the *flavors, servers_specs*, and *servers_utilization* tables are stored in separate CSV files, their shared identifiers enable consistent linking between server observations and the underlying hardware definitions. This integration enables the interpretation of server activity, user behavior, and project analyses in the context of the hardware configurations on which workloads run.

### 3.5 Data gaps

**Fig. 6:**
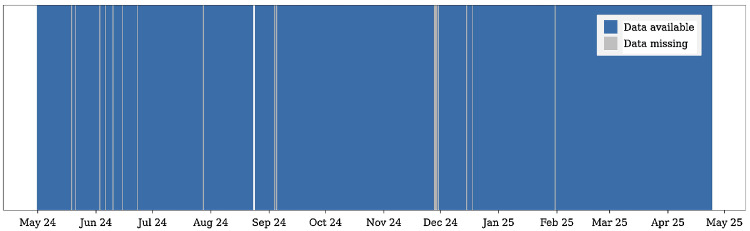
Data availability: Periods with available data are shown in blue, missing data periods in grey, highlighting the overall continuity of the data collection and the presence of occasional gaps.

Fig. 6 shows an overview of data availability and missing periods (gaps) in the *servers_utilization* table, one of the core components of the dataset. Although the collection service was scheduled to record data every five minutes, occasional interruptions were observed. Data gaps were identified when, at a given collection timestamp, all server entries had invalid values for *vcpu_utilization* and *ram_utilization.* Consecutive timestamps meeting this condition were aggregated into contiguous blocks representing periods of missing data.

Given that the collector interacts with OpenStack APIs and Prometheus/libvirt endpoints at five-minute intervals, occasional interruptions or temporary failures in data collection are expected. Nevertheless, these interruptions were generally very short, typically lasting only a few minutes. Overall, periods of missing data represent approximately 0.39 % of the total collection period, indicating that the vast majority of expected measurements are available. Although this analysis focuses on the *servers_utilization.*

### 3.6 Dataset files

**Fig. 7:**
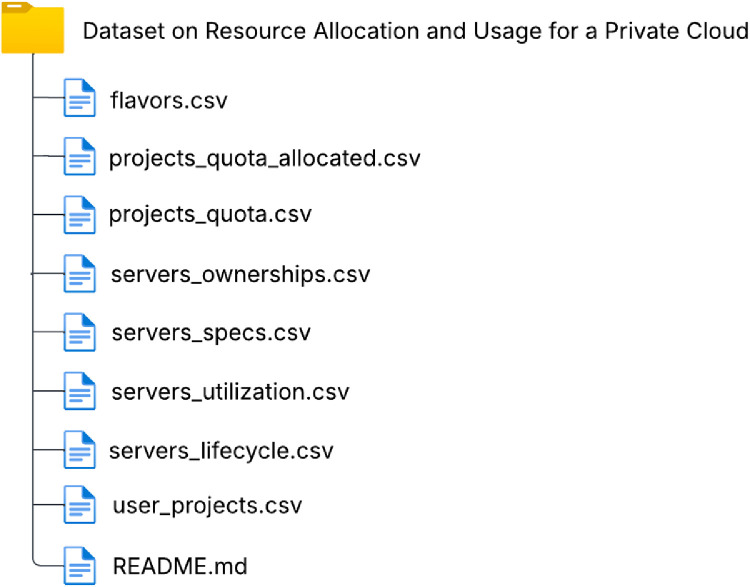
Type of data and dataset files.

The dataset is distributed as a single directory containing 7 CSV files, as shown in Fig. 7. Each file preserves the original schema from the source database, ensuring referential consistency through UUID identifiers (*project_id, user_id, server_id*, and *flavor_id*).

Most tables include a timestamp column indicating the moment of data collection. An exception is *flavors.csv*, which contains static hardware configuration information. The *projects_quota.csv* table also does not contain continuous records; it stores only the moments when quota limits were modified.

Similarly, the *servers_lifecycle* table does not represent a longitudinal time series, but instead records discrete lifecycle events, storing only the creation and termination timestamps of each server. This table complements the periodically sampled tables by providing a complete registry of server lifecycles over the observation period.

A README.md file accompanies the dataset, documenting the structure of the CSV files, the meaning of shared identifiers, and the supported relationships between tables, and providing guidance for combining independent files in exploratory analyses.

## 4 Experimental Design, Materials and Methods

### 4.1 Data collection

The dataset was obtained using a monitoring and data collection tool developed and maintained by the LSD/UFCG. The service runs on a server and periodically queries two main sources:

• OpenStack APIs: used to collect metadata about projects, users, servers, and flavors, including quota definitions and currently allocated resources.
• Prometheus endpoints: provide runtime performance metrics, such as vCPU and RAM utilization, exported via libvirt.

**Fig. 8:**
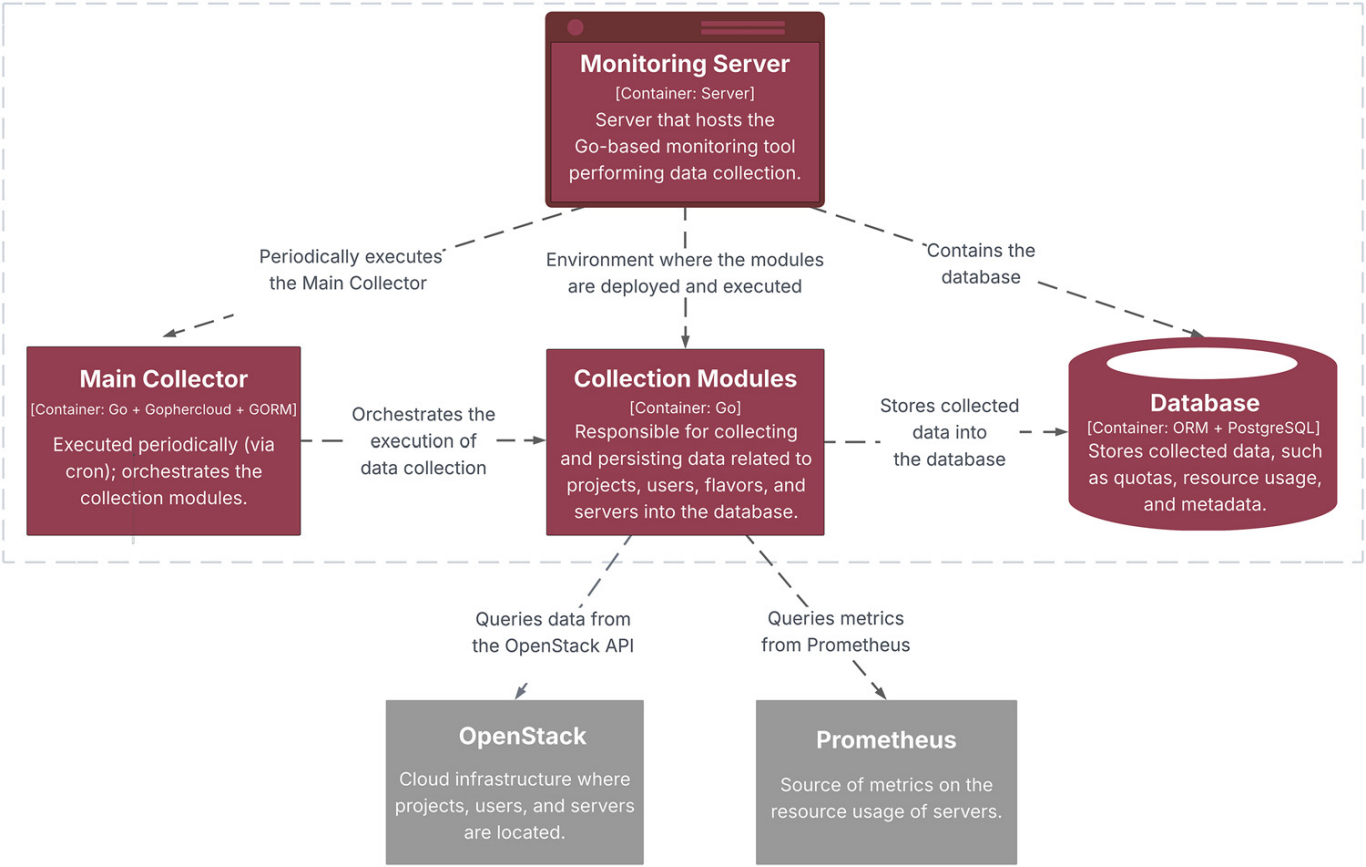
Overview of the data collection and processing workflow.

Collection tasks were scheduled regularly, and the collected data were stored in a PostgreSQL database running on the same server. Fig. 8 illustrates this workflow, in which the monitoring service periodically queries OpenStack and Prometheus for metadata and utilization metrics and persists the collected records in the database. Sample data collection scripts are available at the GitHub repository^1^. The raw data were later extracted using SQL queries.

In addition to this monitoring-based data collection pipeline, the *servers_lifecycle* file was obtained through a separate extraction process by executing direct SQL queries on the production cloud management database, exporting server lifecycle records into a CSV file on January 12, 2026. This file does not contain 5-minute sampled data and instead records server creation and termination events. It is therefore provided as supplementary material to complement the longitudinal server-related tables.

### 4.2 Identifier design and privacy considerations

The dataset relies on the native identification model adopted by OpenStack. In OpenStack, entities such as users, projects, servers, flavors, and hosts are identified using randomly generated UUIDs (UUIDv4), which are created at resource instantiation time and do not encode or derive from any personal or operational attributes. These identifiers are generated using standard UUID generation mechanisms provided by the Python programming language, typically through the uuid.uuid4() function, which produces cryptographically random 128-bit identifiers.

The collection queries omit all columns containing sensitive information, including user, server, and project names, as well as IP addresses. Only the UUIDs automatically generated by OpenStack were retained. As a result, the identifiers act as opaque references for external users, while still enabling consistent cross-table relationships.

### 4.3 Data observation

Data collection was scheduled at fixed five-minute intervals, for over nearly one year of operation. The dataset has >64 million records, covering up to 30 projects. Approximately 160 users were associated with these projects. These users allocated >1193 servers during the observation period. This consistent five-minute sampling, underpins the large volume of time-stamped entries and enables longitudinal analyses of resource behavior within the cloud infrastructure.

To further illustrate this level of granularity, we queried a specific server (*server_id: a23ac1ea-705c-4bd0–9e39-f69bf86f86fd*) over a 30-minute window in May 2024. Table 9 shows the regularity of the five-minute sampling, with each entry reporting vCPU and RAM usage and host identifier.

**Table 9:** Sample vCPU and RAM utilization of a server recorded every 5 min.

timestamp	server_id	vcpu_utilization	ram_utilization	host_id
1716,523,508	a23ac1ea-705c-4bd0…	31.93	16.72	90f4f7e3d4f5bd1f3…
1716,523,810	a23ac1ea-705c-4bd0…	35.13	16.69	90f4f7e3d4f5bd1f3…
1716,524,109	a23ac1ea-705c-4bd0…	34.27	16.70	90f4f7e3d4f5bd1f3…
1716,524,409	a23ac1ea-705c-4bd0…	39.63	16.70	90f4f7e3d4f5bd1f3…
1716,524,709	a23ac1ea-705c-4bd0…	35.43	16.73	90f4f7e3d4f5bd1f3…
1716,525,009	a23ac1ea-705c-4bd0…	33.27	16.66	90f4f7e3d4f5bd1f3…

**Fig. 9:**
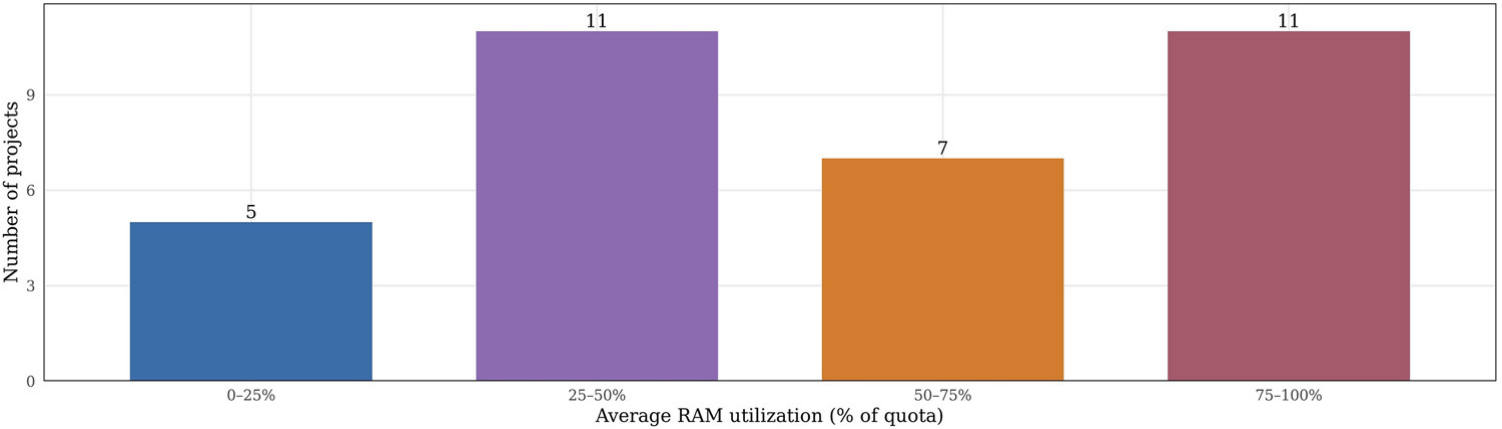
Distribution of projects according to their average RAM utilization relative to allocated quota. Projects are grouped into four utilization ranges: 0–25 %, 25–50 %, 50–75 %, and 75–100 % of their assigned RAM quota. By grouping projects into utilization ranges, the figure illustrates how projects occupy different segments of their assigned capacity, offering a view of utilization distribution across the cloud environment.

Beyond server-level views, the dataset is provided as a collection of CSV files that preserve relationships across files through shared identifiers, while also capturing longitudinal information over the entire observation period (May 2024 to May 2025). Figs. 2, 3, 4, 5, and 9 illustrate these validations, confirming its consistency and ability to capture infrastructure dynamics from multiple perspectives. We provide, in the GitHub repository,^2^ step-by-step instructions for associating the different tables, as well as the R scripts used to generate all figures presented in this article.

To contextualize the scope of the dataset, Table 10 provides a comparison between the data concepts available in this private OpenStack cloud dataset and those typically exposed by publicly available commercial cloud trace datasets. While public traces primarily focus on workload execution and scheduling behavior, this dataset emphasizes infrastructure governance aspects inherent to IaaS environments, including projects, quotas, and server resource utilization.

**Table 10:** Comparative summary of data concepts available in this private OpenStack cloud dataset and in public commercial cloud datasets.

Data concept	This dataset (private OpenStack cloud)	Public commercial cloud datasets
Organizational abstraction	Explicit projects (tenants) grouping users and resources	No explicit tenant abstraction; typically, jobs or services
User representation	Users explicitly modeled and linked to projects	Users represented as anonymized identifiers
Resource limits	Project-level quotas defining enforced allocation limits	Quotas usually not explicitly exposed in traces
Allocation policy context	Allocation and usage can be interpreted under defined quota constraints	Allocation observed without reference to policy-enforced limits
Compute unit	Servers	Tasks, containers, or instances, depending on the dataset
Infrastructure perspective	IaaS governance-oriented view (projects, quotas, ownerships)	Workload execution and scheduling perspective

## Limitations

Although the dataset was collected continuously over approximately a year of operation, there are gaps in the data due to operational and infrastructural factors. These include occasional network instabilities, temporary failures in the server hosting the monitoring tool, and API rate limitations that may have prevented successful data retrieval at specific times. Furthermore, during periods of scheduled maintenance or system upgrades in the cloud infrastructure, the monitoring process may have been paused or partially degraded. These interruptions result in missing entries or uneven sampling intervals in some tables. While such gaps do not significantly compromise the overall representativeness of the dataset, users should be aware of these limitations when conducting time-sensitive analyses or evaluating continuous resource usage patterns.

Because data collection of resource utilization occurs at fixed five-minute intervals, the monitoring service captures only the infrastructure state at each sampling point. Consequently, servers that are created and terminated entirely between two observations do not appear in the dataset. To assess the impact of this sampling interval on the representativeness of the data, we retrieved a server lifecycle log from the cloud management database, which contains the identifier of each server along with its creation and termination timestamps. By cross-referencing this lifecycle information with the monitoring dataset, we identified servers that were dismissed on the resource utilization collection, due to the five-minute collection interval. These missing servers account for approximately 17 % of the total number of instantiated servers. Despite this, the impact on the analyses enabled by the dataset is expected to be minimal, as such servers typically have very short lifetimes and therefore contribute negligibly to overall resource usage patterns. Moreover, a five-minute sampling interval is commonly adopted in comparable cloud monitoring datasets [[1], [2], [3]].

We also acknowledge that retaining persistent UUIDs to enable longitudinal analysis introduces a theoretical risk of re-identification via exploiting side-channel information. We prioritized retaining temporal patterns to facilitate data handling for dataset users.

Finally, because the dataset reflects the operational characteristics of an academic private cloud, its workload dynamics and resource allocation practices may differ from those found in enterprise private environments. The dataset should therefore be interpreted within this scope.

## Ethics Statement

The authors confirm that they have read and follow the ethical requirements for publication in Data in Brief. The current work does not involve human participants, animal experiments, or data collected from social media platforms.

## Credit Author Statement

**Paola Marques:** Writing - Original Draft, Conceptualization, Methodology, Investigation, Data Curation, Visualization; **Mariana Mendes:** Writing - Review & Editing, Visualization; **Thiago Emmanuel Pereira:** Conceptualization, Funding acquisition, Writing - Review & Editing, Supervision**; Giovanni Farias:** Writing - Review & Editing, Project administration.

## Data Availability

Mendeley DataDataset on Resource Allocation and Usage for a Private Cloud (Original data).

## Footnotes

1 https://github.com/ufcg-lsd/private-cloud-data-v2025/tree/main/scripts.

2 https://github.com/ufcg-lsd/private-cloud-data-v2025/tree/main/notebooks.
